# Neutralizing anti-drug antibodies in Fabry disease have no obvious clinical impact?

**DOI:** 10.1186/s13023-018-0916-1

**Published:** 2018-09-29

**Authors:** Malte Lenders, Boris Schmitz, Stefan-Martin Brand, Eva Brand

**Affiliations:** 1 Internal Medicine D, Department of Nephrology, Hypertension and Rheumatology, University Hospital Muenster, Albert-Schweitzer-Campus 1, D-48149 Muenster, Germany; 2 Institute of Sports Medicine, Molecular Genetics of Cardiovascular Disease, University Hospital Muenster, Muenster, Germany

**Keywords:** Enzyme replacement therapy, Longitudinal, Prospective

## Abstract

Fabry disease (FD) is a rare X-linked disorder caused by a deficiency of lysosomal α-galactosidase A activity. Treatment with recombinant enzyme replacement therapy is available since 2001 and the effects of anti-drug antibodies (ADA) on therapy efficacy and disease outcome in affected patients have been controversially reported. In this letter we discuss the importance of adequate measurements of neutralizing ADAs and appropriate longitudinal analysis to determine therapy efficiency and clinical outcome in patients with FD.

## Letter to the editor

With great interest we read the recent paper by Mauhin and colleagues entitled “Deep characterization of the anti-drug antibodies developed in Fabry disease patients, a prospective analysis from the French multicenter cohort FFABRY” [1]. The authors presented a detailed characterization of anti-drug antibodies (ADAs) in patients with Fabry disease, confirmed their frequency of 40% in men treated with agalsidase and complete cross reactivity against agalsidase alfa and beta and an unambiguous association with lyso-Gb3 considered as a hallmark of disease severity [1]. Nevertheless, they came to the conclusion that “anti-agalsidase antibodies have no obvious clinical impact”. By using IgG isotype-specific ELISA-techniques, the authors demonstrated the humoral IgG-dependent response to ERT. They investigated the associations between IgG subtypes and neutralizing activity, and showed that IgG1, IgG2 and IgG4 subtypes were capable of ERT neutralization in vitro. In our opinion to further substantiate these associations it would have been mandatory to use purified IgG subtypes from patients’ sera and test these fractions separately for ERT neutralization as recently demonstrated for IgG4 [2]. This approach would also have been important since it is known that not all ADAs possess neutralizing activity. Thus, ELISA-based determination of an ADA-positive status is not sufficient to define the clinically relevant impact of ERT neutralization. Of note, the clinical use of neutralization assays to assess the effect of ADAs on clinical and biochemical outcome has repeatedly been demonstrated during the last 15 years [2–6]. Future studies need to be performed to analyze a potential impact of neutralizing and non-neutralizing ADAs on endothelial ERT-uptake.

Furthermore, we agree with Mauhin et al. that the presence of planted ERT-ADA immune complexes in kidneys as reported for other lysosomal storage disease (LSDs) [7–9] has not been addressed adequately in recent studies and needs further attention [1]. From the biochemical sight of view the formation of these complexes is probably a rare event, since according to the precipitation curve (Heidelberger curve) of antigen-antibody interaction large non-soluble immune complexes will form when antigens and antibodies are in a balanced state.

In a recent prospective study including longitudinal data, a significant 2.8-fold increased risk for the formation of neutralizing ADA in male patients with FD when treated with agalsidase-beta (1.0 mg/kg every other week) compared to agalsidase-alfa (0.2 mg/kg every other week) was demonstrated [10]. A subgroup analysis of these patients with neutralizing ADAs also revealed a better biochemical response to agalsidase-beta at 1.0 mg/kg in terms of decreasing lyso-Gb3 levels over time [10]. To this respect, we recently demonstrated that antibodies can be saturated and appropriate enzyme dosages can overcome ADA titers during infusions (antigen excess), which resulted in decreased plasma lyso-Gb3 levels [2]. In their cohort, Mauhin and colleagues [1] found no differences between antibody-positive and antibody-negative in non-renal-transplanted patients. Of note, these data are difficult to interpret since the neutralizing impact of the antibodies was not considered, individual slopes were not calculated (only one time-point), and patients with severe renal impairment were excluded from the analysis. The latter point is of specific interest since antibody-positive compared to -negative patients presented with a significantly higher frequency for dialysis and renal transplantation (6 [33.3%] vs 1 [3.7%]; *p* = 0.012) [1].

Moreover, significantly more antibody-positive patients were treated with agalsidase-beta (*n* = 14; 77.8%) than with agalsidase-alfa (*n* = 4; 22.2%; *p* = 0.0012) [1]. In combination with the missing information on ERT-neutralization status, the 5-fold higher dosage due to agalsidase-beta treatment might have led to the observed effect in that no clinical differences between patients with and without ADAs had been observed.

Therefore, we conclude that neutralizing ADAs affect clinical outcomes and independent of the general assay used for neutralizing ADA measurements and titer determination, the adequate longitudinal analysis of the impact of ADAs on therapy efficiency and clinical outcome in patients with FD can only be performed when analyzed patients are stratified according to supersaturated ADA titers during infusion.

## Abbreviations

ADA: anti-drug antibody
eGFR: estimated glomerular filtration rate
ELISA: enzyme-linked immunosorbent assay
ERT: enzyme replacement therapy
FD: Fabry disease
GLA: alpha-galactosidase A
LSD: lysosomal storage disease
